# Phylogenomic analysis of the understudied *Neisseriaceae* species reveals a poly- and paraphyletic *Kingella* genus

**DOI:** 10.1128/spectrum.03123-23

**Published:** 2023-10-26

**Authors:** Daniel P. Morreale, Joseph W. St Geme III, Paul J. Planet

**Affiliations:** 1 Perelman School of Medicine, University of Pennsylvania, Philadelphia, Pennsylvania, USA; 2 Division of Infectious Diseases, Children's Hospital of Philadelphia, Philadelphia, Pennsylvania, USA; 3 Comparative Genomics, American Museum of Natural History, New York, New York, USA; University of Pittsburgh School of Medicine, Pittsburgh, Pennsylvania, USA

**Keywords:** *Kingella*, *Simonsiella*, *Alysiella*, *Neisseriaceae*, polyphyly

## Abstract

Taxonomic classification and phylogenetic analysis of the *Neisseriaceae* family have focused on the pathogens *Neisseria meningitidis* and *Neisseria gonorrhoeae*. Less is known about the relationships of commensal *Neisseria* species and other *Neisseriaceae* genera, raising the possibility that the phylogeny of this family may not agree with current taxonomy. In this study, we used available nucleotide sequences and a phylogenetic approach to assess the *Kingella* genus and its relatives. We found that this genus is both paraphyletic and polyphyletic. *Kingella potus* is more closely related to *Neisseria bacilliformis* than to other *Kingella* species. The *Alysiella* and *Simonsiella* genera form a distinct clade within the *Kingella* genus that is closely related to the pathogens *K. kingae* and *K. negevensis*. We find a phylogenetic relationship between *Conchiformibius*, *Alysiella*, *Simonsiella*, and *Kingella*, which we name the CASK clade. Finally, we define the gene sets that differentiate each genus of the CASK clade from one another and from the rest of the *Neisseriaceae* family.

**IMPORTANCE:**

Understanding the evolutionary relationships between the species in the *Neisseriaceae* family has been a persistent challenge in bacterial systematics due to high recombination rates in these species. Previous studies of this family have focused on *Neisseria meningitidis* and *N. gonorrhoeae*. However, previously understudied *Neisseriaceae* species are gaining new attention, with *Kingella kingae* now recognized as a common human pathogen and with *Alysiella* and *Simonsiella* being unique in the bacterial world as multicellular organisms. A better understanding of the genomic evolution of the *Neisseriaceae* can lead to the identification of specific genes and traits that underlie the remarkable diversity of this family.

## INTRODUCTION

Defining taxonomic relationships is a cornerstone of microbial systematics, but in species that reproduce asexually and trade genetic information through active horizontal gene transfer (HGT) across the species boundary, defining the relationships between species can be challenging. As in many taxonomic disciplines, the relationships among and within species have traditionally been established using bacterial cell morphology and other phenotypic properties. With the advent of molecular techniques such as DNA-DNA hybridization, 16S rRNA gene sequencing, multilocus sequence typing (MLST), and matrix-assisted laser desorption/ionization time of flight (MALDI-TOF), the phenotypic approach gave way to the so-called polyphasic classification strategy that seeks to consider both phenotypic and molecular data (1, 2). With the greater availability of whole-genome sequences, a newer version of this system, taxonomogenomics, seeks to incorporate this rich genomic data into taxonomical descriptions (3 – 6). This approach also has the benefit of being explicitly phylogenetic, providing a non-arbitrary approach to classification (5, 7, 8). While there is still controversy about how best to integrate phenotypic and phylogenomic data in bacterial taxonomy, there is a general consensus that taxonomy should be reflective of the evolutionary relationships and that monophyly is a desirable characteristic of taxonomic groups.

The *Neisseriaceae* family is a group of Gram-negative, β-proteobacteria that currently includes 20 genera: *Neisseria*, *Wielerella*, *Vitreoscilla*, *Uruburuella*, *Stenoxybacter*, *Snodgrassella*, *Simonsiella*, *Rivicola*, *Prolinoborus*, *Paralysiella*, *Morococcus*, *Kingella*, *Eikenella*, *Cronobacter*, *Craterilacuibacter*, *Conchiformibius*, *Bergeriella*, *Aquella*, *Amantichitinum*, and *Alysiella* (9 – 13). The majority of prior work devoted to understanding the taxonomic relationships between these genera has focused primarily on the pathogenic *Neisseria* species, *N. meningitidis* and *N. gonorrhoeae* (2, 8, 14 – 19). Early work using 16S rRNA genes failed to properly resolve species boundaries, resulting in several polyphyletic genera (20). In addition, pervasive HGT and recombination in the *Neisseriaceae* further increase taxonomic ambiguity, with clonal lineages punctuated by large recombination events between distant relatives (16, 21). HGT results in the formation of ill-defined species with ambiguous species boundaries (22). To help resolve this ambiguity, recent studies of the commensal *Neisseria* species employed core genome MLST and proposed genus-specific cutoffs for defining species using genome relatedness indices (8, 21, 22). However, these studies did not comprehensively address other polyphyletic genera within the *Neisseriaceae*.

The *Kingella* genus includes five taxa: *K. potus, K. oralis, K. denitrificans, K. negevensis,* and *K. kingae* (23 – 30). Recently, two novel species have been proposed, namely *K. bonacorsii* and *K. pumchi* (31, 32). Together, these species form a major clade in the *Neisseriaceae* and are most closely related to the genus *Neisseria*. All of the *Kingella* species are typically associated with the oral or oropharyngeal microbiome, a common niche for other *Neisseriaceae*. Isolates of *K. kingae*, *K. negevensis*, and *K. potus* have each been associated with invasive disease in immunocompetent individuals (25, 27, 33). In contrast, *K. oralis* and *K. denitrificans* are present in dental plaque and in gingival and periodontal samples and only rarely cause invasive disease, typically in immunocompromised individuals (28, 34, 35). *Kingella* species are oxidase-positive, catalase-negative rods and typically form short chains (36).

A review of published phylogenetic relationships among the commensal *Neisseria* species reveals a high level of variability in the proposed taxonomic relationships, primarily owing to the genes selected and the method used to perform the analysis (17, 37 – 39). In many of these studies, the *Kingella* genus is polyphyletic, and the genera that interrupt a monophyletic clade with *Kingella* species differ in different published analyses (for examples, see references 26, 36, 37, 40, 41). This variation is dependent on the species the authors chose to include or exclude, the reference isolate used, and the data type used to rebuild the phylogeny. The majority of the isolates used for defining taxonomic nomenclature in this genus were collected prior to pervasive whole-genome sequencing and were assigned to *Kingella* as a result of phenotypic and/or minimal sequence-based analyses. Additionally, as culture-dependent and culture-independent microbiome studies have become more prevalent, the number and diversity of formally accepted species in the *Neisseriaceae* have increased (15). Here, we seek to reassess the relatedness of the five species in the *Kingella* genus and the relationship of the *Kingella* genus to the other genera in the *Neisseriaceae,* using whole genomes and a phylogenomic approach.

## RESULTS

### *Kingella potus* is distinct from the other species in the *Kingella* genus

**Fig 1:**
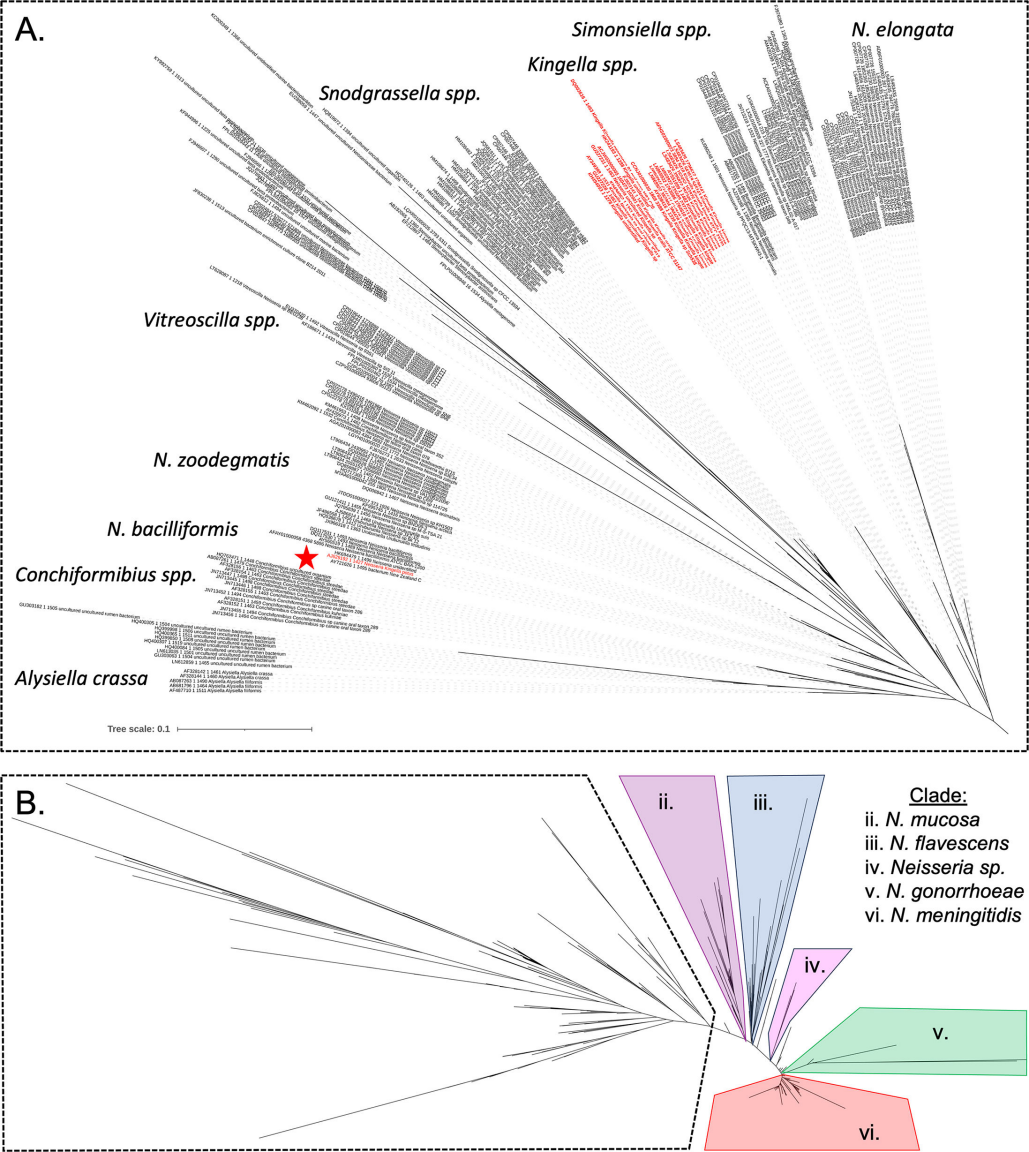
High-quality 16S rRNA gene sequences were downloaded from the SILVA database for the family *Neisseriaceae* (taxid: 408). Sequences were aligned with MAFFT, and a maximum likelihood phylogeny was constructed using RAxML. (A) Genera closely related to *K. kingae* are shown. Species that belong to a species in the genus *Kingella* are colored red. By 16S rRNA relatedness, *Kingella* forms a polyphyletic clade with *K. potus* (red star) falling outside of the rest of the genus. (B) Full phylogeny of all *Neisseriaceae* species in this analysis. The outlined clade appears in panel A.

To examine the *Kingella* genus, 16S rRNA gene sequences for isolates from the *Neisseriaceae* were collected for phylogenetic reconstruction (Fig. 1; Fig. S1). By 16S rRNA gene relatedness alone, it is clear that a major driver of the polyphyletic structure of *Kingella* is *K. potus*. This species was recovered from an infected wound caused by an animal bite and is more closely related to *Neisseria bacilliformis* than it is to any currently described *Kingella* species (27). Phenotypically, *K. potus* and *N. bacilliformis* are defined based on a limited number of characteristics, though both species are non-hemolytic, indole-negative coccobacilli that produce acid from glucose, maltose, and fructose (Table S3).

**Fig 2:**
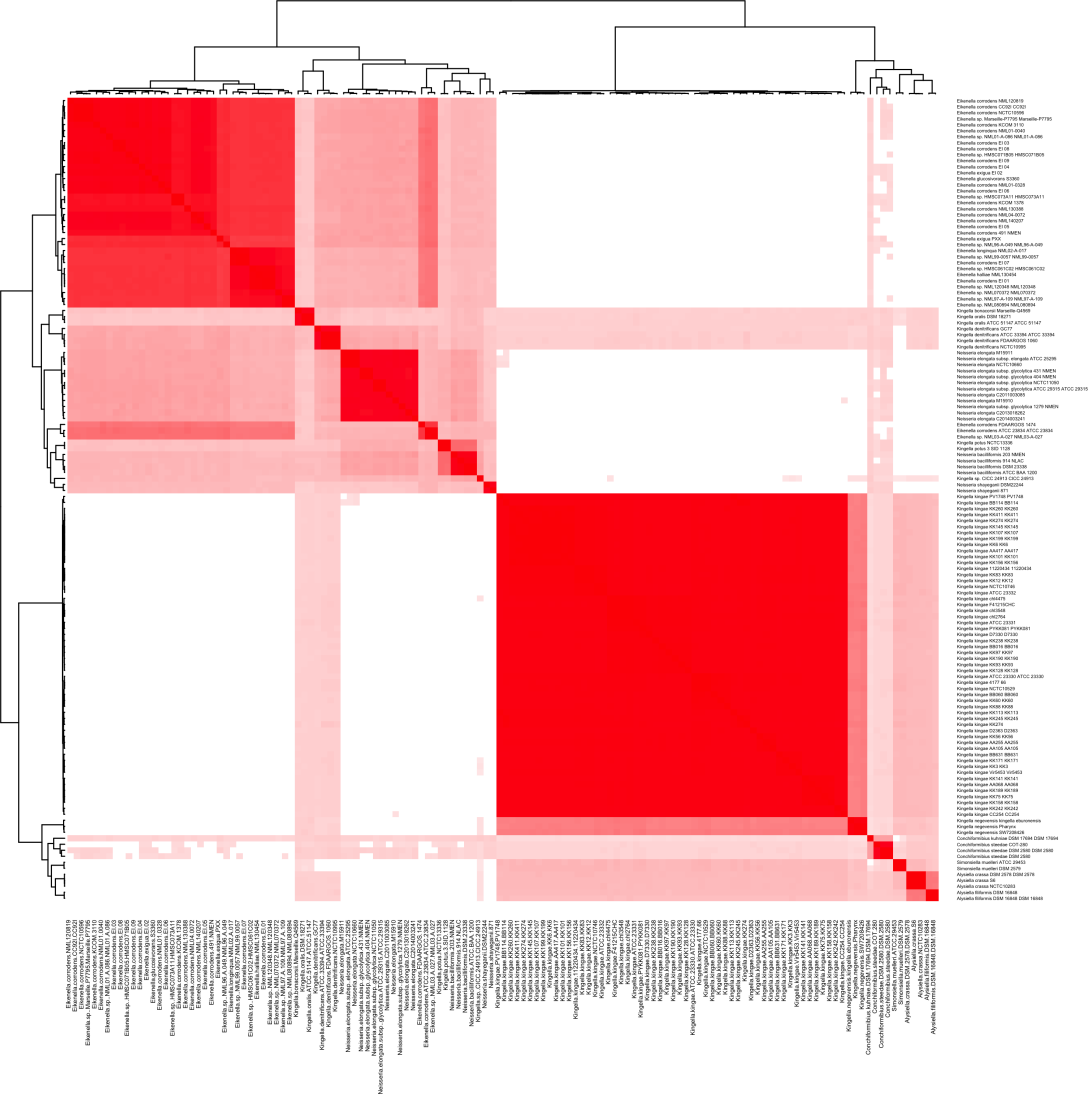
ANI scores comparing sequenced isolates from species in the *Neisseriaceae* most closely related to the genus *Kingella*. ANI scores were calculated between publicly available genomes downloaded from NCBI using FastANI. The resulting ANI matrix was rendered as a heatmap using R and is ordered by the corresponding distance matrix calculated with hierarchical clustering.

To better understand the relationship between *K. potus* and *N. bacilliformis*, we calculated average nucleotide identity (ANI) and digital DNA-DNA hybridization (dDDH) between the type strains of these species. The ANI between *K. potus* and *N. bacilliformis* was calculated at 86.75%, below the established species cutoff of 95% (40). However, the ANI between *K. potus* and *N. bacilliformis* is appreciably greater than the ANI calculated between *K. potus* and other *Kingella* species, which ranges between <75% (*K. kingae*) and 78.8% (*K. denitrificans*) (Table 1; Fig. 2; Fig. S2). The calculated dDDH between these species falls below species thresholds at 30% (95% confidence interval 27.6%–32.5%) (Table 2).

**TABLE 1:** Average ANI scores calculated among taxa of interest^*a*^

	*N.* *bacilliformis*	*K.* *denitrificans*	*K.* *oralis*	*K.* *negevensis*	*K.* *kingae*	*N.* *elongata*	*A.* *crassa*	*A.* *filiformis*	*S.* *muelleri*
*K. potus*	86.75	78.88	78.74	<75	<75	80.7	–	–	–
*K. bonacorsii*	–	79.84	96.41	78.06	77.9	78.76	77.98	77.87	77.14
*A. crassa*	–	77.81	77.96	79.37	79.74	<75	100	85.43	79.79
*A. filiformis*	–	77.81	77.96	79.37	79.74	<75	85.43	100	78.75
*S. muelleri*	–	<75	76.9	79.16	79.96	<75	79.79	78.75	100

^*a*^ ANI scores were calculated using FastANI and are shown only if the ANI >75%. Dashes denote data excluded from the table.

**TABLE 2:** dDDH values of *K. potus* calculated against closely related taxa^*a*^

Query strain	Subject strain	dDDH (d0 [%])	95% confidence interval (d0 [%])	dDDH (d4 [%])	95% confidence interval (d4 [%])	dDDH (d6 [%])	95% confidence interval (d6 [%])	G+C content difference (%)
*K. potus* NCTC13336	*N. bacilliformis* ATCC BAA-1200	45.6	42.2–49.0	30	27.6–32.5	41	38.0–44.0	1.86
*K. potus* NCTC13336	*N. elongata* subsp. *glycolytica* ATCC 29315	20.3	17.1–23.9	22.5	20.2–25.0	19.7	17.0–22.8	3.71
*K. potus* NCTC13336	*K. negevensis* Sch538	12.9	10.2–16.2	23.5	21.2–25.9	13.3	10.9–16.0	12.24

^*a*^ dDDH was calculated using TYGS using each of the possible algorithms, as well as the difference in G+C content between *K. potus* and each of the subject-type strains.

### The *Kingella* genus is paraphyletic

To further define the relationships among *Kingella* and closely related species, whole-genome sequences for select species in the *Neisseriaceae* were downloaded from NCBI. As 16S rRNA gene sequences have been historically used to define species in this group, all the following analyses were limited to species most closely related to *Kingella* by 16S rRNA gene phylogeny (Fig. 1). This group includes *Neisseria elongata, Neisseria sheyganii, Neisseria bacilliformis, Eikenella corrodens, Alysiella filiformis, Alysiella crassa, Simonsiella muelleri, Conchiformibius steedae, K. oralis, K. bonacorsii, K. potus, K. denitrificans, K. kingae,* and *K. negevensis. Neisseria elongata*, *Neisseria sheyganii,* and *Eikenella corrodens* were included as outgroups for these analyses. In total, 134 whole-genome sequences were downloaded from NCBI and annotated with Prokka.

**Fig 3:**
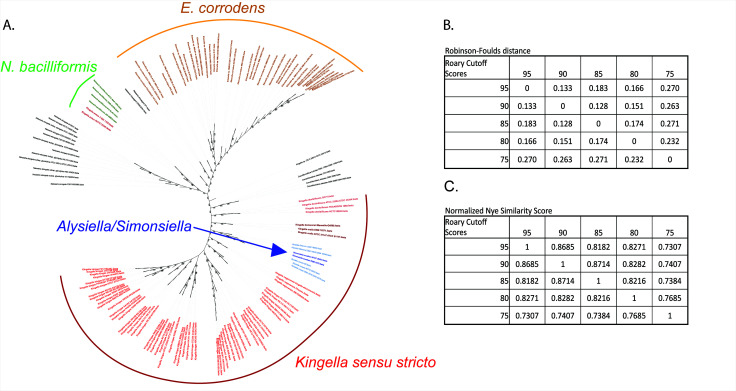
(A) The core genome of the strains from the 13 species most closely related to *Kingella kingae* was determined using Roary with a minimum similarity cutoff of 75%. The core genomes, which encompass approximately two million sites, were aligned with MAFFT and used to reconstruct a maximum likelihood phylogeny. As above, *K. potus* is only distantly related to the rest of the species in the genus *Kingella*. Additionally, the genera *Alysiella* and *Simonsiella* fall within the *Kingella* clade, suggesting the core genome of the species in each of these genera is very closely related to that of *Kingella*. Bootstraps greater than 70% are shown. (**B–C)** The Robinson-Foulds and normalized Nye similarity score congruency metrics were used to quantify changes in tree architecture between phylogenies reconstructed using core genome alignments. The Nye similarity score was normalized against the number of possible rearrangements given the number of leaves in the tree.

To minimize the impact of HGT and variability in the pangenome, Roary was used to identify the core genome (41). Core genes were clustered at the 75%, 80%, 85%, 90%, and 95% sequence similarity level and were limited to only those clusters of orthologous groups (COGs) found in all genomes. Core gene alignments were generated and used to reconstruct maximum likelihood phylogenies (Fig. 3). Both the Robinson-Foulds distance and normalized Nye similarity metrics were used to quantify tree similarity. Each phylogeny was highly congruent with the others based on these metrics, and each included a polyphyletic clade for the genus *Kingella* (Fig. 3B and C). We, therefore, proceeded with the lowest similarity cutoff of 75% for further analyses (Fig. 3A). In each of these analyses, *Conchiformibius* spp., *Alysiella* spp., *Simonsiella* spp., and *Kingella* spp. (with the exception of *K. potus*) form a closely related group that we will refer to as the CASK clade. This clade defines a major group within the *Neisseriaceae*. Phenotypic data on the species in the CASK clade are extremely limited, reducing the utility of phenotypic properties in understanding relationships within the group (Table S3).

To ensure that this group is not an artifact as a result of selecting these species, we repeated this analysis using representative isolates from every species in the *Neisseriaceae*. Whole-genome sequences for up to 10 isolates of each species were collected from the GenBank database (586 total strains, Table S2). Core genes were defined with Roary. To account for the increase in diversity, core genes were defined as occurring in at least 80% of isolates and possessing at least 50% sequence similarity, further reducing stringency. A codon-aware alignment was performed on the core genome, and a tree was reconstructed based on 192,906 sites using IQ-Tree (Fig. S3). Even in this less selective analysis, we found that the CASK clade remains intact and forms a monophyletic group distinct from the remainder of the *Neisseriaceae*. Moreover, we found that *Kingella* remains paraphyletic and that *Wielerella bovis* is incorporated into this group.

Based on both whole-genome approaches, *Alysiella* and *Simonsiella* form a clade within the *Kingella* genus (Fig. 3A), dividing *K. oralis* and *K. denitrificans* from *K. negevensis* and *K. kingae*. ANI, dDDH, and G+C% were calculated for type strains of *Kingella*, *Alysiella*, and *Simonsiella* and are shown in Tables 1 and 3. All of these overall genome relatedness indices (OGRI) support a close relationship between the species of these three genera.

**TABLE 3:** OGRI of *Kingella*, *Alysiella*, and *Simonsiella^a^*

Query strain	Subject strain	dDDH (d0 [%])	95% confidence interval (d0 [%])	dDDH (d4 [%])	95% confidence interval (d4 [%])	dDDH (d6 [%])	95% confidence interval (d6 [%])	G+C content difference (%)
*A. crassa* NCTC10283	*A. filiformis* DSM 16848	21.7	18.5–25.3	34.4	31.9–36.9	21.9	19.1–25.0	1.29
*A. crassa* NCTC10283	*W. bovis* CCUG 44,465T	18.4	15.3–22.0	33.4	31.0–35.9	18.8	16.1–21.8	2.67
*A. crassa* NCTC10283	*S. muelleri* ATCC 29453	16.9	13.8–20.4	23.2	20.9–25.6	16.9	14.3–19.8	3.83
*A. crassa* NCTC10283	*K. kingae* ATCC 23330	16.3	13.3–19.8	23.8	21.5–26.3	16.4	13.8–19.3	1.45
*A. crassa* NCTC10283	*K. negevensis* Sch538	15.6	12.7–19.0	23.3	21.0–25.8	15.7	13.2–18.6	0.17
*A. crassa* NCTC10283	*K. bonacorsii* Marseille-Q4569	14.2	11.4–17.6	22.7	20.5–25.2	14.5	12.0–17.3	8.71
*A. crassa* NCTC10283	*K. oralis* ATCC 51147	14.2	11.4–17.6	22	19.7–24.4	14.5	12.1–17.3	9.01
*A. filiformis* DSM 16848	*A. crassa* NCTC10283	21.8	18.6–25.5	34.1	31.7–36.6	22	19.2–25.1	1.25
*A. filiformis* DSM 16848	*W. bovis* CCUG 44,465T	17.2	14.2–20.8	30.5	28.1–33.0	17.5	14.9–20.5	3.95
*A. filiformis* DSM 16848	*K. kingae* ATCC 23330	16.5	13.5–20.0	24	21.7–26.5	16.5	14.0–19.5	0.17
*A. filiformis* DSM 16848	*K. negevensis* Sch538	15.1	12.2–18.6	24.1	21.8–26.6	15.3	12.8–18.2	1.11
*A. filiformis* DSM 16848	*S. muelleri* ATCC 29453	15.1	12.2–18.6	23.7	21.4–26.1	15.3	12.8–18.2	5.12
*A. filiformis* DSM 16848	*C. steedae* DSM 2580	15.1	12.2–18.5	22.6	20.3–25.0	15.3	12.8–18.2	4.17
*A. filiformis* DSM 16848	*K. bonacorsii* Marseille-Q4569	14.6	11.7–18.0	22.8	20.5–25.2	14.8	12.3–17.6	7.43
*A. filiformis* DSM 16848	*K. oralis* ATCC 51147	14.6	11.8–18.0	22.2	19.9–24.7	14.8	12.4–17.7	7.72

^*a*^ dDDH was calculated using TYGS using each of the possible algorithms, as well as the difference in G+C content between type strains of *Alysiella* or *Simonsiella* species and each of the closest related subject-type strains.

Additionally, we calculated pairwise ANI scores for each of the genomes listed above. The ANI scores confirm the majority of the proposed species classifications except for *K. bonacorsii*, an unclassified *Kingella* isolate that was recently proposed to be a new species (31). This isolate was recovered from a gingival sample, and the authors reported a 16S rRNA similarity of 98.7%, ANI of 95.83%, and dDDH of 63.6% relative to *K. oralis* UB-38, as well as non-identification by MALDI-TOF mass spectrometry (31). We replicated each of the genomic calculations reported as well as additional dDDH calculations using alternative formulae and found that choice of formula is essential, as only *d*4 yielded a dDDH below the accepted values for species cutoffs (Tables 1 and 4; Fig. 2) (40, 42).

**TABLE 4:** COGs responsible for the unification of *Kingella s.s*. in the accessory genome

	COG	State	p_0^*a*^	p_1^*b*^		State	p_0^*a*^	p_1^*b*^	COG name^*c*^	Predicted function^*c*^
Last common ancestor *Alysiella, Simonsiella,* and *Kingella s.s*.^*d*^	742	1	0.15414	0.84586	Last common ancestor of *Kingella s.s*.	0	0.98197	0.01803	group_2532	Protein IscX
	766	1	0.02993	0.97007		0	0.97924	0.02076	group_2994	Hypothetical protein
	771	1	0.02993	0.97007		0	0.97924	0.02076	*purU*	Hypothetical protein
	923	1	0.24673	0.75327		0	0.97821	0.02179	group_3650	Sulfur carrier protein TusA
	3159	1	0.06887	0.93113		0	0.96883	0.03117	group_6852	Isocitrate lyase
	3277	1	0.35969	0.64031		0	0.96819	0.03181	*thiG*	Thiazole synthase
	3284	1	0.35969	0.64031		0	0.96819	0.03181	group_5996	Imidazole glycerol phosphate synthase subunit HisH
	3586	1	0.13975	0.86025		0	0.95545	0.04455	group_5655	Isocitrate dehydrogenase (NADP)

^*a*^ Maximum likelihood posterior probability that this COG was absent at this site in the inferred ancestral reconstruction. Genes were called as absent if p_0 ≥0.95.

^*b*^ Maximum likelihood posterior probability that this COG was present at this site in the inferred ancestral reconstruction. Genes were called as present if p_0 ≥0.95.

^*c*^ Predicted names and functions of COGs of interest were recovered from the associated Roary matrix. Sequential COGs are not necessarily syntenic in any given genome.

^*d*^ *Kingella s.s*., *Kingella sensu stricto.*

### *Kingella* species are unified by gene presence/absence in the pangenome

**Fig 4:**
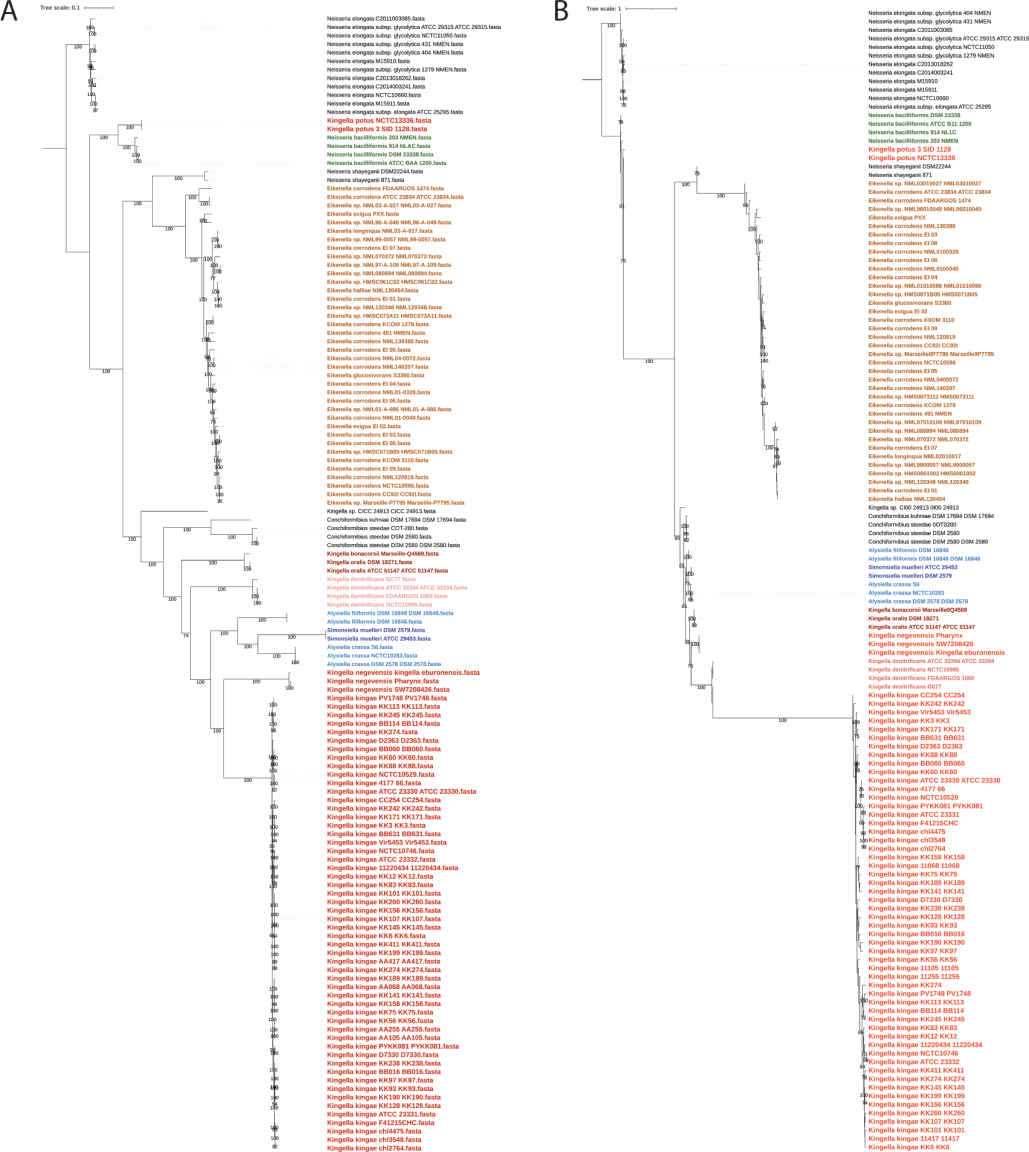
(A) The core genome of the strains from the 13 species most closely related to *Kingella kingae* was determined using Roary with a minimum homology cutoff of 50% with COGs present in at least 80% of the species. The core genomes, which encompass approximately two million sites, were aligned with MAFFT and used to reconstruct a maximum likelihood phylogeny. As above, *K. potus* is only distantly related to the rest of the species in the genus *Kingella*. Additionally, the genera *Alysiella* and *Simonsiella* fall within the *Kingella* clade, suggesting the core genome of the species in each of these genera is very closely related to that of *Kingella*. Bootstraps greater than 70% are shown. The phylogeny is rooted by the *Neisseria elongata* clade. (**B)** A binary gene presence/absence matrix was generated using Roary at the 75% similarity cutoff and includes both the core and accessory genes of all species. A phylogenetic tree based on this matrix was constructed using RAxML via the GAMMA model of rate heterogeneity. This analysis, based solely on gene content unites *Kingella sensu stricto* into a monophyletic group. Bootstraps greater than 70% are shown. The phylogeny is rooted by the *Neisseria elongata* clade.

Another recent evolutionary study of these taxa has used less stringent similarity and gene presence cutoffs to define the core genome for species in the *Neisseriaceae* for subsequent phylogenetic analyses (37). We replicated this analysis by reanalyzing the data using a sequence similarity cutoff of 50% for genes present in 80% of analyzed genomes. By lowering the threshold for a gene to be considered part of the core genome, we increased the number of genes considered from 109 at the 75% similarity level up to 620 genes at the new, less stringent cutoff. Notably, as we increased the number of genes considered part of the core genome by decreasing the stringency of our Roary analysis, the presence of a polyphyletic *Kingella* clade remains constant (Fig. 4A), demonstrating the robust relationships between these genera that are obtained when we consider the core genome of species in the *Neisseriaceae*.

To better understand how the pangenome impacts the relationships described in the above analyses, we reconstructed the phylogeny considering only gene presence and absence in the pangenome. This analysis was performed with a binary COG presence/absence matrix as defined by Roary with a 75% similarity cutoff with phylogenetic inference in IQ-Tree. For the first time in any of our analyses, all named *Kingella* species except for *K. potus* are monophyletic, excluding the *Simonsiella* and *Alysiella* genomes. To identify genes that drive the unification of *Kingella sensu stricto* (*Kingella s.s*.), IQ-Tree was used to perform ancestral reconstructions of the species in this tree. These ancestral reconstructions facilitate the identification of the genes that drive the unification of *Kingella* into a monophyletic clade. Discounting statistically ambiguous sites, monophyly is driven by the loss of eight genes in *Kingella s.s*. (Table 4; Table S10), all of which fall in the accessory genome. There are no gene gains that support this clade, and therefore, this monophyly is not well supported.

These data further suggest that HGT can confound our ability to define species relationships and that limits on HGT may be important to consider when defining species in the *Neisseriaceae*. HGT is driven by DNA uptake sequences (DUS) in the *Neisseriaceae* (43 – 45), and these sequences are largely thought to be species specific. The DUS in the CASK clade have been previously characterized (43). Type genomes for each species were used to define DUS preference by word search. *Alysiella filliformis*, *Alysiella crassa*, *Simonsiella muelleri*, and *Kingella oralis* genomes are enriched for the AG-SimDUS (AGGCTGCCTGAA) and the AG-KingDUS (AGGCAGCCTGAA). *Kingella kingae, Kingella negevensis,* and *Kingella denitrificans* genomes are enriched for the AA-King3DUS (AAGCAGCCTGCA). *Conchiformibius steedae* and *Conchiformibius kuhniae* do not appear to use any of these previously defined DUS.

### The CASK species are unique based on cellular morphology and lifestyle

To identify the genes that differentiate the species in the CASK clade and to define COGs unique to groups of interest, Scoary was used to analyze the pangenome analysis generated by Roary at the 75% sequence similarity level. The presence of genes is defined as diagnostic for the in-group if they are 100% specific, are 100% sensitive, and have *P* < 0.001. These cutoffs reduce the impact of shared mobile genetic elements (MGEs) such as prophages or genomic islands.

**Fig 5:**
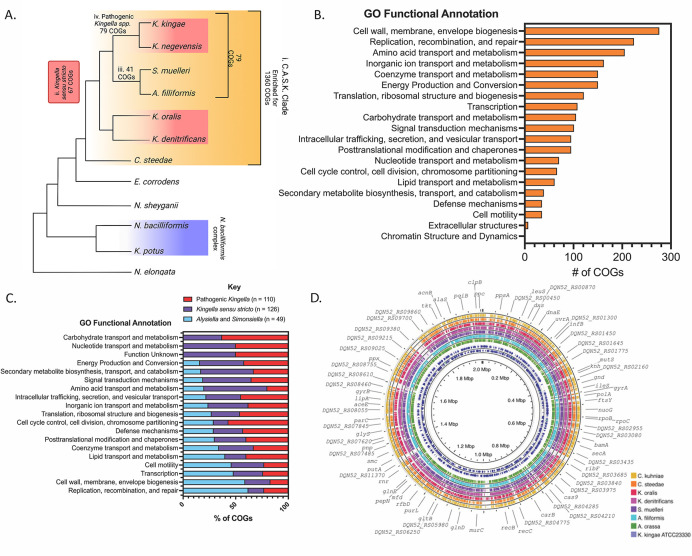
(A) Scoary was used to generate lists of differentially encoded genes comparing (i) CASK species against the *Neisseriaceae*, (ii) *Kingella sensu stricto,* (iii) *Alysiella* and *Simonsiella,* or (iii) pathogenic *Kingella* against the CASK clade. Finally, *Alysiella* and *Simonsiella* COGs were compared against the pathogenic *Kingella*. Diagnostic COGs are defined as COGs with 100% sensitivity and specificity to the in-group and filtered to *P < 0.001*. Please see Tables S5 to S9 list for full COG sets.** (B–C)** Diagnostic gene sets for each group were annotated using eggNOG mapper and classified by Gene Ontology functional groups using the NCBI COG database. (B) The CASK clade is enriched for unique COGs with putative roles in cell wall, membrane, and envelope biogenesis, as well as DNA replication, recombination, and repair. COGs with no functional annotations were omitted from this graphic (*n* = 656 COGs).** (C)** Similarly, the majority of the unique COGs present in *Alysiella* and *Simonsiella* may function as part of DNA replication, recombination, and repair pathways and cell wall, membrane, and envelope biogenesis. *Kingella sensu stricto* and the pathogenic *Kingella* species encode more unique COGs implicated in carbohydrate, amino acid, or nucleotide transport and metabolism.** (D)** Type strains of each species in the CASK clade was compared to the *K. kingae* ATCC 23330 genome (inner circle) using BLASTn. Bars in each ring represent regions of sequence similarity between genomes. Each of the CASK species shows significant sequence similarity to nearly all of the *K. kingae* reference, further demonstrating how closely related each of these species are. Regions of dissimilarly map to regions that encode putative MGEs, particularly prophages.

First, the CASK clade was compared against the rest of the species included in the phylogenetic analyses (Fig. 5A; Table S5). This clade is enriched for 1,360 COGs that are absent in the rest of the analyzed species, providing strong evidence supporting the evolutionary relatedness of this group. The average genome size for the species in this group is approximately 2,000 coding sequences. To understand putative roles of these COGs in the CASK species, COG groups and Gene Ontology functional annotations were defined with eggnog mapper (Fig. 5B). Approximately half of the COGs were assigned to the “function unknown” group (*n* = 656). Of the remaining COGs, the majority play putative roles in cell wall, membrane, and envelope biogenesis (*n* = 276); DNA replication, recombination, and repair (*n* = 224); or amino acid transport and metabolism (*n* = 205).

Next, *Kingella s.s.*, comprising *K. oralis, K. denitrificans, K. negevensis,* and *K. kingae*, was compared to the rest of the CASK clade. Only 67 COGs meet the criteria to be diagnostic for *Kingella s.s.* (Table S6). When *Alysiella* and *Simonsiella* or the pathogenic *Kingella* spp. were compared to the rest of CASK clade, we identified only 41 or 79 diagnostic COGs, respectively (Fig. 5A; Tables S7 and S8). Again, we assigned putative functional roles for the COGs in each of these groups (Fig. 5C; Table S9). The COGs diagnostic for *Kingella s.s*. and the pathogenic *Kingella* spp. (*K. negevensis* and *K. kingae*) are primarily involved in carbohydrate transport and metabolism or nucleotide transport and metabolism. By contrast, *Alysiella* and *Simonsiella* diagnostic COGs are involved in DNA replication, recombination, and repair or cell wall, membrane, and envelope biogenesis (Fig. 5C).

Finally, we compared the genomes of the type strain for each species in the CASK clade using BLASTn against *K. kingae* ATCC 23330 (Fig. 5D). We found high levels of nucleotide sequence similarity across the *K. kingae* genome relative to the related species. There are few notable regions of dissimilarity, which appear to be MGEs in the *K. kingae* genome.

## DISCUSSION

Classification of the species in the *Neisseriaceae* has been difficult using traditional metrics of relatedness, both phenotypically and phylogenetically. As a result, the family has been restructured several times since its inception to include or exclude genera (17, 26, 39, 46, 47). Species in this family resist robust phylogenetic classification due to pervasive HGT and recombination, including in the 16S rRNA sequence (20 – 22, 43). Due to the large diversity of species and variability, phenotypic classification using strict phenotypic classification is either difficult or impossible without additional sampling. For example, presence of catalase is conserved among *Neisseria* isolates, and yet isolates of *N. elongata* have been recovered that lack the catalase enzyme (36). Furthermore, as more species are proposed using culture-independent methods or based on a single isolate, it will be essential to establish phylogenomic species definitions for the *Neisseriaceae* (1, 20, 22).

In this study, we employed a phylogenetic approach to define the evolutionary relationships within a major subgroup of the *Neisseriaceae*, defined by the genera *Eikenella, Conchiformibius, Alysiella, Simonsiella, Kingella*, and select *Neisseria* species. Examination by 16S rRNA gene sequence unveiled a very recent common ancestor between *K. potus* and *N. bacilliformis*, which was further supported by several other genome relatedness indices (Fig. 1; Tables 1 and 3). *K. potus* is a catalase-negative species collected from an infected wound that resulted from a bite by a captive South American Kinkajou (*Potus flavus*) (27). *N. bacilliformis* is an opportunistic pathogen that has been recovered from human infections, varies in the production of catalase (48), and is phenotypically identical to *N. elongata* subsp. *elongata.* These species were reported nearly simultaneously, preventing their direct comparison. Our reanalysis suggests that *K. potus* is much more closely related to *N. bacilliformis* than to any other *Kingella* species, though it is likely a distinct species from *N. bacilliformis*. Inclusion in the genus *Kingella* was likely due to a negative catalase test, underscoring the importance of a genomic approach to classification of these isolates.

The phylogenomic analyses also uncovered close evolutionary relationships between *Conchiformibius* sp., *Alysiella* sp.*, Simonsiella* sp., and *Kingella* sp.*,* which we have named the “CASK” clade (Fig. 3). The CASK clade is a major branch of the *Neisseriaceae* that includes both invasive pathogens (*K. negevensis* and *K. kingae*) and oral-associated commensals that are distinct from *Neisseria* spp. *Alysiella* and *Simonsiella* are genera associated with the oral microbiome of mammals (17, 46, 49 – 52). *Conchiformibius* spp., *Alysiella* spp., and *Simonsiella* spp. are remarkable for their multicellularity, which is the result of incomplete septation by forming the peptidoglycan during bacterial division (37).

Reanalysis with an expanded data set suggests the close relationship between *Wielerella bovis* and the CASK species. Only recently described, *W. bovis* was isolated post mortem from cattle suffering from endocarditis in two European countries (47). Little is known regarding the pathogenesis and carriage of this species among cattle or other animals, and the species has yet to be recovered from the oral-associated microbiome of cattle. However, like many members of the commensal *Neisseriaceae*, significant work is still required to understand the carriage and disease incidence of this species.

The reproducibility of the core genome phylogenetic structure very strongly supports the close relationship between the species of the CASK clade and the exclusion of *K. potus* (Fig. 3 and 4). This relationship is maintained through regardless of the definition used to define the core genome of the analyzed isolates. The phylogeny reconstructed from a core genome defined as genes present in 100% of isolates with 95% sequence similarity (3 genes, 2,133 sites) is congruent with the phylogeny reconstructed from less stringent analyses. These results suggest that the taxonomic classification of proposed isolates belonging to the *Neisseriaceae* could be improved with even small amounts of sequencing data for *rplT, atpD*, and *rpsL*.

We hypothesized that prior misclassification could be due to high levels of HGT of accessory genes between *Kingella* (*sensu stricto*) genomes. To test this hypothesis, we generated a presence/absence matrix generated by Roary at 75% sequence similarity level, a technique that emphasizes accessory genes over core genes, and we reconstructed a phylogeny using the RAxML binary model with gamma model of rate heterogeneity and bootstrapped 100 times (Fig. 4B). This was the only analysis in which we observed a monophyletic *Kingella* genus, though *Kingella potus* was still excluded. We favor the analyses that are based on nucleotide sequence of the core genome because they contain much more genomic information (~76,500 variable sites compared to ~29,000 variable genes) and likely lessen confounding signal from recombined areas.

A separate whole gene-based analysis also identified more diagnostic genes uniting *Alysiella*, *Simonsiella,* and the pathogenic *Kingella* species (*n* = 79) than genes uniting the *Kingella* genus *sensu stricto* (*n* = 69). Additionally, *Simonsiella*, *K. oralis*, and *K. kingae* utilize specific DNA uptake sequences, suggesting that they may be able to share DNA more easily among themselves, as compared to the rest of the *Neisseriaceae*. Given that members of the *Neisseriaceae* are highly recombinogenic and undergo significant HGT of accessory genes, we propose that the core genome is a higher fidelity evolutionary record for this family (22).

The CASK clade has a large set of diagnostic genes that differentiate these species from the remainder of the *Neisseriaceae*. Interestingly, this group is enriched for processes that impact cell wall and envelope biogenesis (Fig. 5B), likely driven by the unusual cellular morphologies of *Conchiformibius* spp., *Alysiella* spp., and *Simonsiella* spp. These genera are distinctive for being multicellular, longitudinally dividing (MuLDi) species that comprise the only known animal multicellular bacterial symbionts (37). Nyongesa et al. hypothesize that the MuLDi phenotype evolved twice in the *Neisseriaceae,* first in *Conchiformibius* species and then in the common ancestor of *Alysiella* and *Simonsiella*, a hypothesis that is strongly supported by our phylogenetic analyses. This hypothesis is further supported by consideration of the diagnostic genes that differentiate *Alysiella* and *Simonsiella* from the rest of this group (Fig. 5C). We again find an enrichment for COGs implicated in cellular morphology, as we would expect if these genera evolved the MuLDi phenotype independently of *Conchiformibius* spp. By contrast, *Kingella s.s.* is enriched for COGs associated with nutrient transport and metabolism (Fig. 5C).

Our study suggests that the current taxonomic classifications of species in the *Kingella* genus may not reflect evolutionary history. Based on our results, *K. potus* is more related to *N. bacilliformis* than to other *Kingella* species by 16S rRNA genes, OGRI, and whole-genome analyses and should likely be assigned to the genus *Neisseria* or another genus entirely. Additionally, phylogenomic analysis of the core genomes of *Alysiella* and *Simonsiella* indicates their close relationship to the *Kingella* genus and supports either their reassignment to *Kingella*, resulting in a monophyletic genus, or the removal of *K. denitrificans* and *K. oralis* from this genus.

## MATERIALS AND METHODS

### Phylogenetic analysis

Of the available 16S rRNA gene sequences for isolates from the *Neisseriaceae* and in the RefNR database, 840 were downloaded from the SILVA r138.1 database on 8 September 2022 (53, 54). 16S sequence accession numbers are listed in Table S1. 16S rRNA sequences were aligned with MAFFT, and a phylogeny was reconstructed using RAxML v. 8.4.2 with the general time reversible model of nucleotide substitution and gamma model of rate heterogeneity, and bootstrapped 100 times (55, 56).

Whole-genome sequences for *Neisseria elongata, Neisseria sheyganii, N. bacilliformis, Eikenella corrodens, Alysiella filiformis, Alysiella crassa, Simonsiella muelleri, Conchiformibius steedae, K. oralis, K. bonacorsii, K. potus, K. denitrificans, K. kingae,* and *K. negevensis* were downloaded from the NCBI Genome database on 9 September 2022. Sequence accession numbers are listed in Table S2. In total, 134 genomes were reannotated with Prokka v1.14.6, using default settings for bacterial sequences (57). Phenotypic characteristics for each of these species were collected by literature review and are listed in Table S3. Assemblies were analyzed with Roary v3.13.0. to identify the core genome (41). Core genes were clustered at the 75%, 80%, 85%, 90%, and 95% sequence similarity level and were limited to only those clusters of orthologous groups found in all genomes (-cd 100), unless otherwise noted. Additionally, other recent evolutionary study of these taxa has used less stringent similarity and gene presence cutoff to define the core genome for species in the *Neisseriaceae* for subsequent phylogenetic analyses (8, 37). We replicated this analysis by reanalyzing the data using a sequence similarity cutoff of 50% for genes present in 99% or 80% of analyzed genomes. Nucleotide sequences of core genes were compared in a codon-aware alignment by PRANK within Roary, and core gene alignments were used to reconstruct phylogenies using RAxML as above. Phylogenetic trees were annotated using Figtree v.1.4.4 and ITOL (58). Bootstrap values >70 are shown.

Additionally, for each species in the *Neisseriaceae,* up to 15 isolates were selected at random from the GenBank database. For species with less than 10 sequenced isolates, all available sequencing was downloaded. This resulted in a total of 586 genomes. Genomes were annotated with Prokka v1.14.6, using default settings for bacterial sequences (57). Assemblies were analyzed with Roary v3.13.0. to identify the core genome (41). Core genes were clustered with a minimum sequence similarity cutoff of 50% for genes present in 80% of analyzed genomes. Nucleotide sequences of core genes were compared in a codon-aware alignment by PRANK within Roary, and core gene alignments were used to reconstruct phylogenies using the General Time Reversible model with ascertainment bias and 1,000 Ultrafast Bootstraps in IQ-Tree v. 2.0.3 (59 – 61). Phylogenetic trees were annotated using Figtree v.1.4.4 and ITOL (58). To calculate phylogenetic tree similarity, final trees were analyzed in R using the Ape v5.6-2 (62), ggtree v3.1.2 (63), and TreeDist v.2.5.0 (64, 65) packages . The Robinson-Fould distance and normalized Nye similarity score were determined for each pair of trees (64 – 67).

Ancestral reconstructions were performed using the Roary gene presence/absence (PA) matrix generated at the 75% sequence similarity cutoff. The matrix was converted into a FASTA formatted file of binary data using R. This file was used for phylogenetic reconstruction in IQ-Tree v 1.6.12 with automatic model calling (60, 61). Branch support was determined via ultrafast bootstrapping (-bb 1000) (59), and ancestral states (-asr) were calculated with default cutoffs. Full inferred ancestral state matrices are available in Supplementary File 2. To determine the COGs that unite *Kingella s.s.*, the inferred PA matrix of common ancestor of *Alysiella* spp., *Simonsiella* spp., and *Kingella s.s.* (Node 7) was compared to the inferred PA matrix of common ancestor of *Kingella* spp. Statistically ambiguous sites present in either PA matrix were removed from this analysis.

### Gene set enrichment

Scoary v 1.6.16 was used to further analyze the gene presence/absence matrix generated by Roary at the 75% sequence similarity cutoff (68). The -r flag was used to limit the set of strains considered in this analysis. COGs were eliminated from this analysis if the Bonferroni-corrected *P*-value >0.0001. Diagnostic genes are only those genes which (i) meet this statistical threshold, (ii) are enriched in the clade of interest, and (iii) have specificity and sensitivity of >80%.

To identify pathways that are enriched in each phylogenetic group, the representative COG sequence was collected from the Roary output files and compiled into a gene set. Gene sets were analyzed with the COG database using eggNOG-mapper v 2.1.9 (69, 70), with a DIAMOND alignment (71) and default settings for bacterial species (Tables S4 to S9). Results were plotted using Graphpad Prism.

### Genome relatedness indices

Average nucleotide identity and digital DNA-DNA hybridization between the type strains of these species were calculated using FastANI v.1.33 and the Type Strain Genome Server (TYGS), respectively (72 – 74). A heatmap of ANI scores was drawn using ggplot heatmap.2 and with hierarchical distance clustering in R.

## Supplementary Material

Reviewer comments

## Data Availability

Newick formatted phylogenetic trees, as well as all custom scripts, are available at https://zenodo.org/badge/latestdoi/697872093. All whole-genome sequences were downloaded from NCBI Genbank database. 16S rRNA sequences were downloaded from the SILVA database.

## SUPPLEMENTAL MATERIAL

Supplemental figures spectrum.03123-23-s0001.pdf Fig. S1 to S3.

Supplemental tables spectrum.03123-23-s0002.xlsx Tables S1 to S10.

OPEN PEER REVIEW reviewer-comments.pdf An accounting of the reviewer comments and feedback.

ASM does not own the copyrights to Supplemental Material that may be linked to, or accessed through, an article. The authors have granted ASM a non-exclusive, world-wide license to publish the Supplemental Material files. Please contact the corresponding author directly for reuse.
