# Supplementary material for: Phylogenomic analysis of the understudied Neisseriaceae species reveals a poly- and paraphyletic Kingella genus
Source: Microbiol Spectr. 2023 Oct 26;11(6):e03123-23. doi: 10.1128/spectrum.03123-23 (PMC10715097; doi:10.1128/spectrum.03123-23)
Supplement: Supplemental figures — Fig. S1 to S3. [file spectrum.03123-23-s0001.pdf]

Supplementary Figures

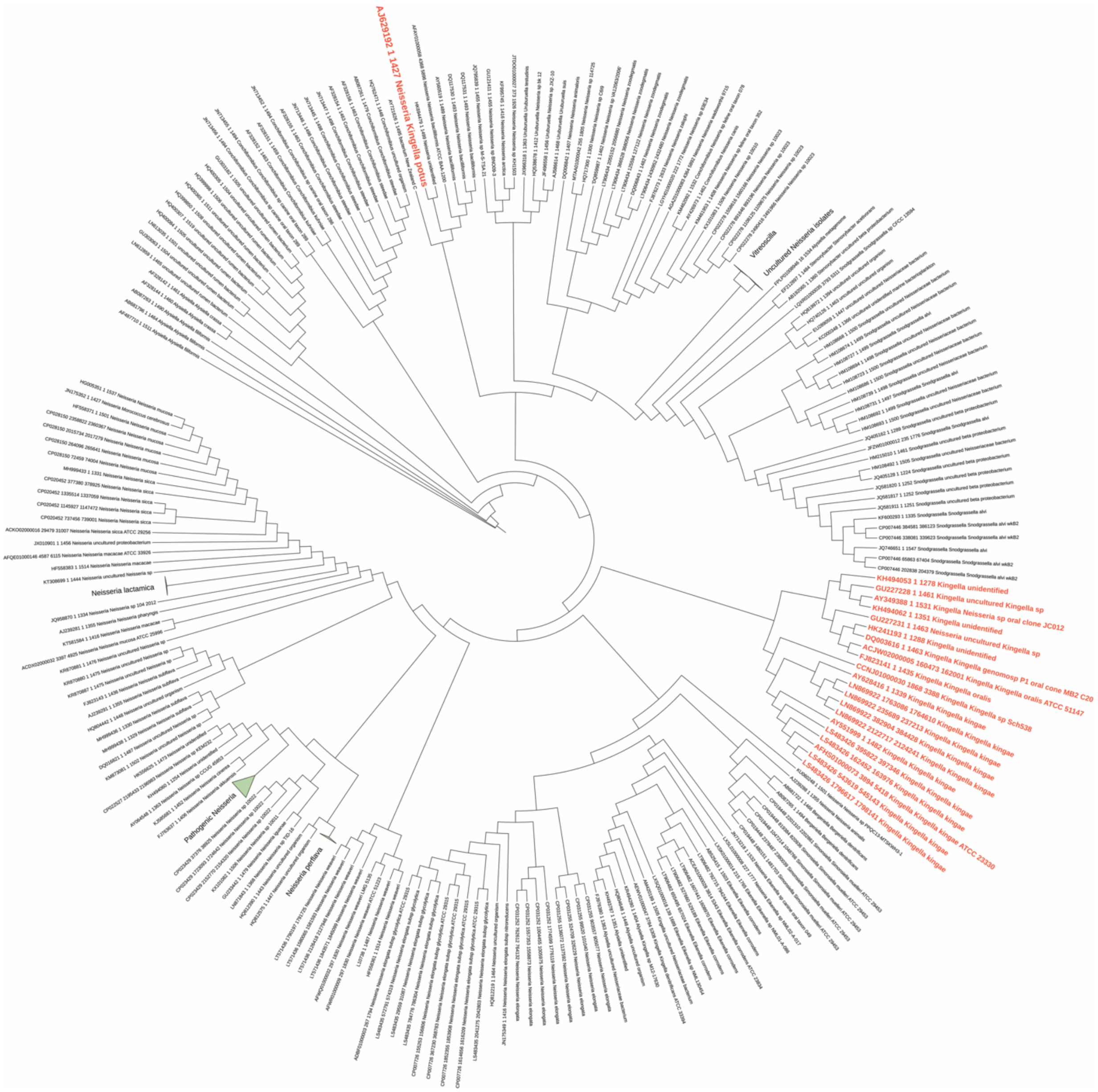

Figure S1. High quality 16S rRNA gene sequences were downloaded from the SILVA database for the family *Neisseriaceae* (taxid: 408). Sequences were aligned with MAFFT, and a maximum likelihood phylogeny was constructed using RAxML. The phylogeny was visualized as a cladogram to better demonstrate polyphyly in the genus *Kingella*.

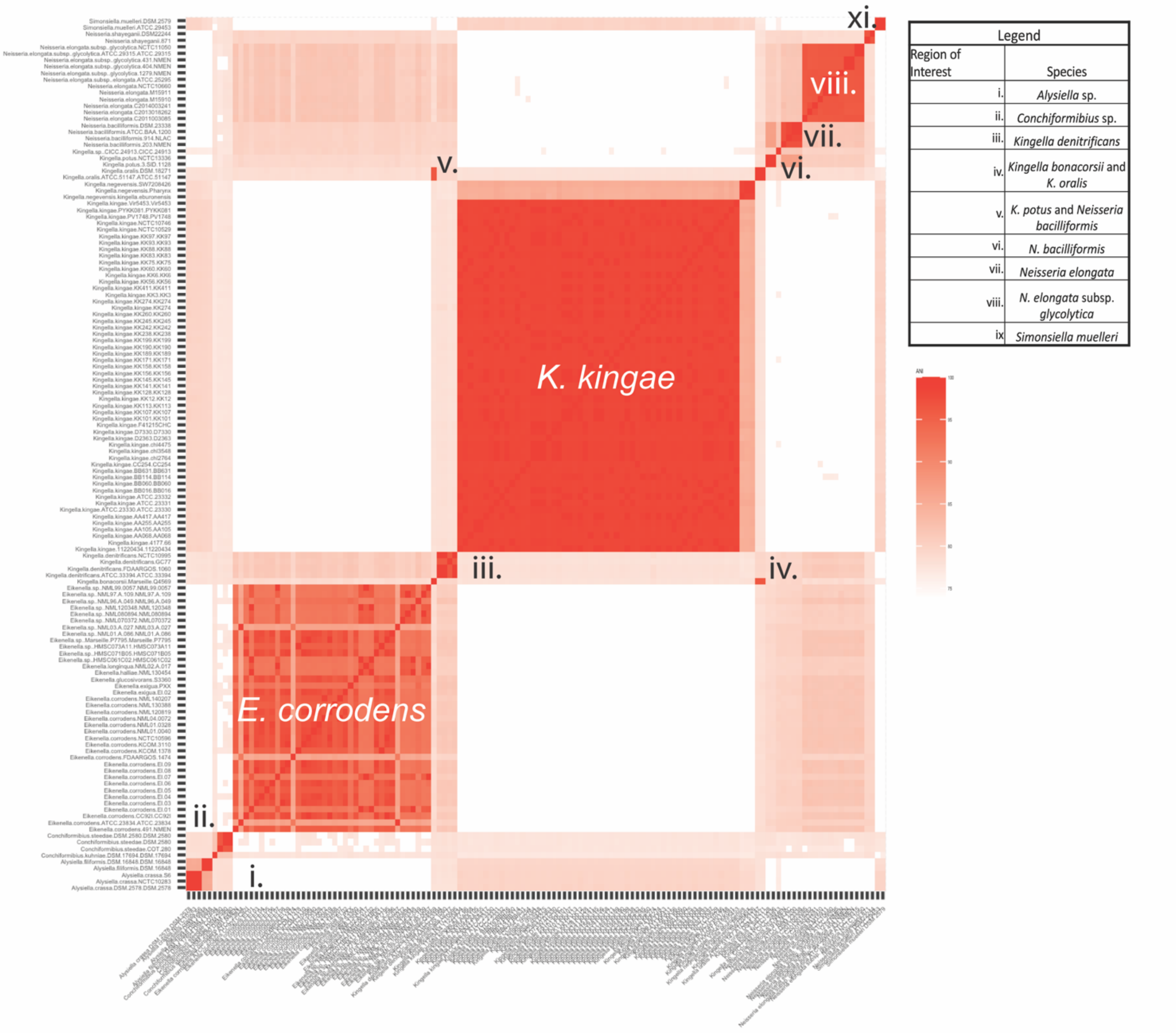

Figure S2. ANI scores comparing sequenced isolates from species in the *Neisseriaceae* most closely related to the genus *Kingella*. ANI scores were calculated between publicly available genomes downloaded from NCBI using FastANI. Several regions of interest are indicated on the heatmap, including the species delimiters, and regions of interest comparing *K. bonacorsii* and *K. oralis* (iv) and *K. potus* and *N. bacilliformis* (v). The resulting ANI matrix was rendered as a heatmap using R and is ordered to reflect the current taxonomic classifications of analyzed isolates.

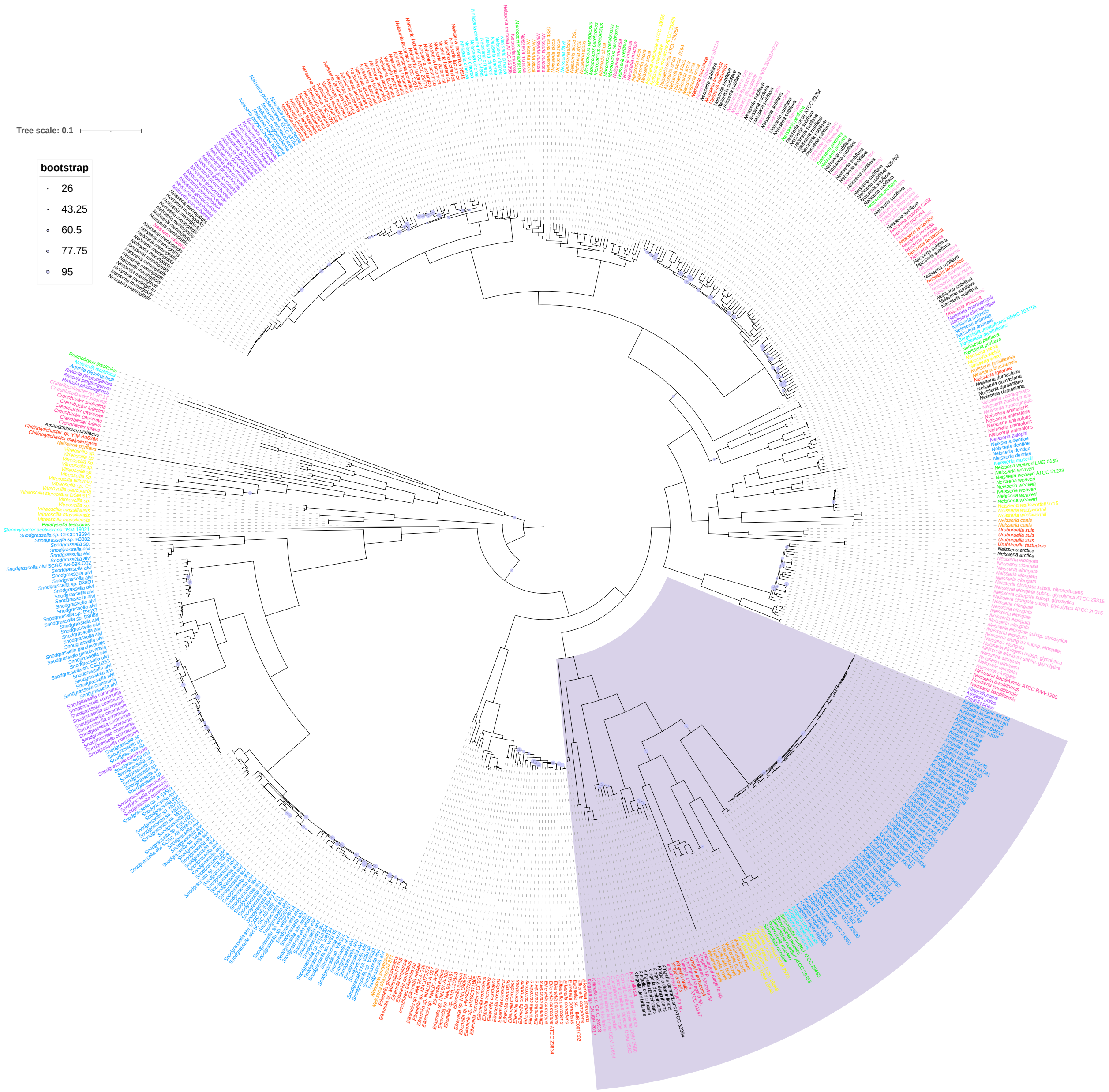

Fig S3. For each species and subspecies in the *Neisseriaceae,* up to 15 isolates were selected at random from the GenBank database. For species with less than 10 sequenced isolates, all available isolates were downloaded, for a total of 586 genomes. Assemblies were analyzed with Roary v3.13.0. to identify the core genome (38). Core genes were clustered with a minimum sequence similarity cut off of 50% for genes present in 80% of analyzed genomes, and a core gene

alignment was used to reconstruct a phylogenetic tree. Branch labels are colored by species. Purple highlighting denotes the CASK clade of the *Neisseriaceae*.
